# ACE Inhibitor Induced Isolated Angioedema of the Small Bowel: A Rare Complication of a Common Medication

**DOI:** 10.1155/2021/8853755

**Published:** 2021-02-13

**Authors:** Marc D. Squillante, Anna Trujillo, Joseph Norton, Saurabh Bansal, David Dragoo

**Affiliations:** ^1^Department of Emergency Medicine, University of Illinois College of Medicine-Peoria, 530 NE Glen Oak Avenue, Peoria, Illinois 61637, USA; ^2^Emergency Medicine Residency Program, St. Mary Mercy Hospital, 36475 Five Mile Rd., Livonia, MI 48154, USA; ^3^Department of Internal Medicine, University of Illinois College of Medicine-Peoria, 530 NE Glen Oak Avenue, Peoria, Illinois 61637, USA; ^4^Department of Radiology, University of Illinois College of Medicine-Peoria, 530 NE Glen Oak Avenue, Peoria, Illinois 61637, USA

## Abstract

Angioedema is a subcutaneous or submucosal tissue swelling due to capillary leakage and transudation of fluid into the interstitial tissue. It can be localized or generalized as part of a widespread reaction known as anaphylaxis. Millions of people in United States and all over the world receive ACEI antihypertensive therapy. ACEI is known to cause angioedema with an incidence of 0.7 percent. We present a case of 40-year-old female who was started on lisinopril three days prior to presentation for newly diagnosed hypertension. She presented with nonspecific severe abdominal pain, nausea, and vomiting. She denied having difficulty breathing or swelling anywhere in the body. On exam, she did not have facial, lip, tongue, or throat swelling. Her abdomen was tender without guarding or rigidity. Laboratory examination was unrevealing except for mild leukocytosis. Computed tomography scan (CT scan) of the abdomen with oral and IV contrast revealed a moderate amount of ascites with diffuse wall thickening, hyperenhancement, and mucosal edema of the entire small bowel. In the absence of any other pathology, matching history, and imaging findings highly suggestive of angioedema, she was diagnosed with isolated small bowel angioedema as a result of ACEI therapy. She was managed conservatively, and lisinopril was discontinued. A week later on follow-up, all her symptoms had resolved, and repeat CT scan showed resolution of all findings.

## 1. Introduction

Millions of people in the United States receive antihypertensive therapy. Angiotensin-converting enzyme inhibitors (ACEI) such as lisinopril and fosinopril are one of the most commonly prescribed agents to treat hypertension [1]. Angioedema due to ACEI, mediated by bradykinin, is a rare but known side effect of this therapy. Most patients present with contiguous organ involvement such as the lips, mouth, tongue, and throat [2]. Angioedema involving the throat or upper respiratory tract is considered a medical emergency. Diagnosis is relatively quick based on visual appearance. Herein, we report a rare case of isolated small bowel angioedema developing three days after initiating ACEI therapy.

## 2. Case Description

**Figure 1:**
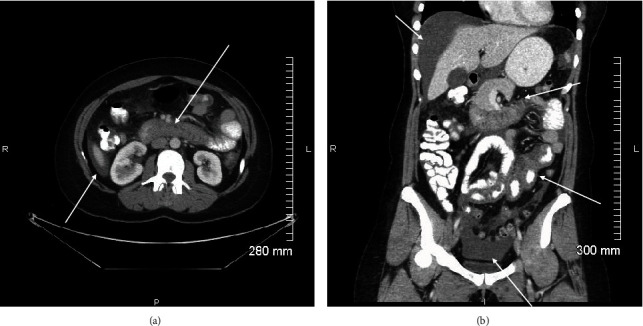
Axial (a) and coronal (b) contrast-enhanced CT scan of the abdomen and pelvis. Images demonstrate a moderate amount of abdominopelvic ascites with diffuse wall thickening, hyperenhancement, and mucosal edema of the entire small bowel.

A 40-year-old Caucasian female presented to the emergency department with diffuse abdominal pain, nausea, emesis, and bloating for the previous 24 hours. She denied any pruritus or rash. She had been to a different ED two days prior with chest pain, abdominal pain, and nausea. An unremarkable cardiac work-up was performed, and the patient was discharged in stable condition. Her medical history included irritable bowel syndrome and newly diagnosed hypertension. Her daily medications included lisinopril 20 mg daily, which she had begun taking 3 days prior, and an oral contraceptive. Vital signs were unremarkable, and she was afebrile. On physical exam, no oropharyngeal or facial swelling was noted. There was moderate generalized abdominal tenderness without rebound, guarding, or rigidity. The remainder of her exam, including her skin, was unremarkable. Her complete blood count showed a mild leukocytosis of 13,000, but all other labs including electrolytes, liver functions, urinalysis, lipase, lactic acid, and troponin were unremarkable. EKG and chest X-ray were both within normal limits. The patient was given intravenous (IV) fluids and hydromorphone for pain. She continued to have diffuse abdominal pain and tenderness. A computed tomography scan of the abdomen with oral and IV contrast was obtained, which revealed a moderate amount of ascites with diffuse wall thickening, hyperenhancement, and mucosal edema of the entire small bowel (Figures 1(a) and 1(b)). Angioedema of the small intestine due to ACE-inhibitor use was the primary differential. The patient was admitted for observation and further evaluation. Lisinopril was discontinued. Gastroenterology was consulted and agreed with the diagnosis and management. The patient's symptoms gradually resolved with supportive care after discontinuation of the lisinopril. A hereditary angioedema work-up was performed which showed normal complement and C1 esterase inhibitor levels. The patient was discharged one day later, and a repeat computed tomography scan performed one week later demonstrated complete resolution of her visceral angioedema and near complete resolution of the ascites.

## 3. Discussion

Angioedema is a subcutaneous or submucosal tissue swelling due to capillary leakage and transudation of fluid into the interstitial tissue. It can be localized or generalized as part of a widespread reaction known as anaphylaxis [3]. Triggering allergens can be found in foods, medications, chemicals, or other substances. Angioedema does not follow the Law of Starling's forces and commonly accumulates in nongravity-dependent areas, in a nonpitting fashion, thus differentiating it from edema due to heart failure or similar conditions. Angioedema is generally benign and transient but can be life-threatening when it causes airway compromise involving the lips, tongue, uvula, pharynx, or larynx [3]. The most common mechanism resulting in angioedema is antigen-induced which is mast-cell histamine-mediated. Generally, these reactions are associated with pruritus, flushing, and urticaria. It is a type-1 immunologic reaction involving IgE consistent with immediate-type hypersensitivity. Nonsteroidal anti-inflammatory agents (NSAIDs) are the most common drugs that result in angioedema due to this mechanism [3].

The second less common mechanism is bradykinin mediated, where previous antigen exposure is not required. Angiotensin-converting enzyme inhibitor- (ACEI-) induced angioedema is a common example where bradykinin excess due to reduced degradation results in angioedema. Such reactions are slow in onset, not associated with pruritus or urticaria, and can take days to develop, thus making them more difficult to diagnose [2, 3].

ACEI are the leading cause of angioedema due to drugs in the United States. The incidence is 0.7% in the first five years of use; 0.07% develop it within the first month. Half of the cases develop within the first week of ACEI therapy. It is five times more common in African Americans than Caucasians [2]. Over 75 million adults in the United States have hypertension, and approximately seventy percent are treated with antihypertensive medication. Among those receiving treatment, at least one in three receive a prescription for an ACEI during their lifetime. ACEI are also prescribed for other indications such as congestive heart failure, diabetes mellitus, and proteinuria. Despite an overall low incidence of angioedema, due to the large number of people taking this class of medications, one can imagine many thousands of cases of angioedema in any given year, and it underscores the fact that it is likely underreported [1, 4].

Angioedema due to ACEI can develop shortly after starting the medications or after many years of use. It is not dose dependent. ACEI result in reduced degradation of bradykinin resulting in elevated levels of bradykinin in the upper respiratory and gastrointestinal tracts. Bradykinin is known to result in vasodilation and increased vascular permeability. Angiotensin II receptor blockers (ARBs) do not affect kinin metabolism directly but are known to cause angioedema. One large retrospective registry did not find an increased incidence of angioedema in patients treated with ARBs who had a history of ACEI-associated angioedema [5].

The differential diagnosis for these patients is broad and involves any type of colitis such as infectious, inflammatory, ischemic, or microscopic. Gastroenteritis (viral or bacterial), radiation enteritis, pancreatitis, hypoalbuminemia, and trauma are also in the differential. Mild elevation in the white blood cell count can occur, but evaluation for infectious etiologies usually is negative. If the ascitic fluid is withdrawn and sent for analysis, it generally shows an elevated serum to ascites albumin gradient as well as reactive mesothelial cells on cytology [6, 7].

History is extremely important in such patients, especially inquiring about exposure to any antigens. A detailed medication history, including prescribed or over-the-counter agents must be obtained. A history of recurrent episodes should always be elicited, as it may indicate C1 inhibitor deficiency. If C1 deficiency is suspected, then C4 and C1 inhibitor antigen levels and functional essays for C1 esterase inhibitor activity should be obtained. Baseline C4 levels could be a good starting point before ordering extensive antigen assays. Reportedly, bowel wall edema is more common with C1 inhibitor deficiency whether hereditary or acquired. Therefore, in cases presenting with bowel wall angioedema alone, it should be ruled out with appropriate testing [3]. In our patient, availability of a detailed history that the patient was recently started on an ACEI allowed the radiologist to make this diagnosis on imaging with high certainty.

Of note, bowel angioedema may self-resolve even when the ACEI is not discontinued, and future recurrences could be severe and more frequent. Alternatively, recurrent episodes of bowel angioedema may occur in the first months even after stopping the ACE inhibitor, and patients should be informed of this possibility. No obvious risk factors have been identified, but occurrence rates are higher in females and African American patients [8].

### 3.1. Imaging

Given that the clinical presentation and symptoms of ACEI-induced angioedema of the small bowel may mimic other acute abdominal pathology, imaging—in particular, contrasted CT of the abdomen and pelvis—is frequently obtained. Imaging findings of small bowel angioedema on contrasted CT of the abdomen and pelvis include diffuse thickening of the small bowel wall and mucosa, mucosal hyperenhancement, fluid distention of the small bowel, engorgement of the mesenteric vasculature, and ascites [7]. Contrasted CT is able to delineate the marked edema within the submucosal layer as a low attenuation separating the muscularis and serosa from the avidly enhancing mucosa (Figures 1(a) and 1(b)). Mesenteric ischemia can be ruled out with patent vessels on contrast-enhanced computed tomography. While imaging in and of itself is not specific for the diagnosis of ACEI-induced small bowel angioedema, when combined with the proper clinical scenario, a high degree of diagnostic certainty can be achieved, preventing unnecessary surgical exploration of the abdomen. Once the inciting ACEI is discontinued, clinical symptoms abate and imaging of the abdomen will return to normal [6, 7, 9].

### 3.2. Management

Initial management includes close monitoring for airway compromise and early endotracheal intubation in patients with impending respiratory failure. Fluid resuscitation, removal of the offending agent, and other supportive measures should be instituted immediately. Epinephrine, antihistamines, or glucocorticoids do not have a major role in treatment as they do not reverse the underlying pathophysiology.

Diagnostic laparoscopy has no role in making this diagnosis but has been utilized in many case reports due to diagnostic uncertainty, adding to the morbidity of the patient [10, 11]. Ultrasound has limited value due to the broad differential diagnosis and need for more detailed imaging in such cases. Computed tomography findings along with a readily available adequate history in the correct context can guide the radiologist and the treatment team in making an accurate diagnosis. With the absence of emergent signs, diagnostic laparoscopy and other invasive testing must be avoided [6, 7].

Treatment is supportive, and if the ACE inhibitor has not been stopped, then a recurrence of angioedema is found to be higher in such patients [12]. Other therapies such as the bradykinin antagonist icatibant and fresh frozen plasma have shown mixed results in studies. They can be used in progressively life-threatening situations, with the caveat being that they should be given within the first few hours of symptom onset [13, 14, 15]. The patient should be advised to avoid taking any ACE inhibitors in the future.

## 4. Conclusion

We present a patient with abdominal pain diagnosed with small bowel angioedema due to recently initiated ACE inhibitor therapy. A detailed medication history (even if the patient has been taking an ACEI for months to years), along with typical CT scan findings, can help the clinician make the correct diagnosis and avoid more invasive therapies (i.e., surgery). Discontinuing the offending ACEI and close monitoring usually result in rapid resolution of symptoms. Avoiding further use of ACEI is important to prevent recurrences.

## Data Availability

None to report.
